# Prospective phase II trial of first-line rituximab, methotrexate, and orelabrutinib (R-MO) in primary central nervous system lymphoma

**DOI:** 10.1038/s41408-025-01278-w

**Published:** 2025-04-29

**Authors:** Lixia Sheng, Hailing Liu, Xiaohui Zhang, Kaiyang Ding, Jie Ma, Hongling Peng, Xia Zhao, Mei Sun, Wei Shi, Feiyan Zhang, Jianyong Li, Lei Cao, Lei Fan

**Affiliations:** 1 Department of Hematology, the First Affiliated Hospital of Ningbo University, Ningbo, 315010 Zhejiang China; 2 Department of Hematology, Jiangsu Province Hospital, the First Affiliated Hospital with Nanjing Medical University, Nanjing, 210029 Jiangsu China; 3 Jiangsu Key Lab of Cancer Biomarkers, Prevention and Treatment, Collaborative Innovation Center for Personalized Cancer Medicine, Nanjing Medical University, Nanjing, 210029 Jiangsu China; 4 Department of Hematology, the Second Affiliated Hospital of Soochow University, Suzhou, 215004 Jiangsu China; 5 Department of Hematology, Anhui Provincial Hospital, the First Affiliated Hospital of USTC, Hefei, 230002 Anhui China; 6 Department of Hematology, the First Affiliated Hospital of Zhengzhou University, Zhengzhou, 450052 Henan China; 7 Department of Hematology, the Second Xiangya Hospital, Central South University, Changsha, 410011 Hunan China; 8 Department of Lymphoma, the Affiliated Hospital of Qingdao University, Qingdao, 266555 Shandong China; 9 Department of Hematology, Northern Jiangsu People’s Hospital Affiliated to Yangzhou University, Yangzhou, 225003 Jiangsu China; 10 Department of Hematology, the Friendship Hospital of Ili Kazakh Autonomous Prefecture, Yining, 835000 Xinjiang China

**Keywords:** Cancer immunotherapy, B-cell lymphoma

## Abstract

The treatment of primary central nervous system lymphoma (PCNSL) is currently limited by the impermeability of the blood-brain barrier. This study aims to assess the efficacy and safety of the R-MO regimen (rituximab, high-dose methotrexate, and orelabrutinib) in the treatment of patients with newly diagnosed PCNSL. A total of 37 patients were enrolled in this prospective, multi-center phase II trial. The post-induction overall response rate (ORR) was 90.3%, and the complete response rate (CRR) was 87.1%. Throughout the trial, the best ORR was 97.1%, and the best CRR was 94.1%. With a median follow-up of 12.6 months, the median progression-free survival (PFS) was not reached, with a 1-year PFS rate of 83.6%, meeting the primary study endpoint. The 1-year overall survival rate was 89.6%. Notably, there was no significant difference in PFS between transplanted and non-transplanted groups (*P* = 0.226). The most common adverse events were neutropenia, lymphocytopenia, and infections, each occurring in 45.9% of patients. Overall, the addition of orelabrutinib to high-dose methotrexate and rituximab in newly diagnosed PCNSL patients has demonstrated promising outcomes and favorable safety profiles, advocating for the use of this combination therapy as a potential frontline treatment option for PCNSL.

## Introduction

Primary central nervous system lymphoma (PCNSL) is a highly aggressive non-Hodgkin lymphoma confined to the central nervous system (CNS) or eyes [1]. Its incidence has increased over the past two decades, reaching 0.47 cases per 100,000 person-years [2]. Approximately 90% of PCNSL cases share the same pathologic features as diffuse large B-cell lymphoma (DLBCL); however, PCNSL faces unique challenges compared to systemic DLBCL, particularly regarding drug penetration across the blood-brain barrier (BBB) [3].

There is currently no consensus on the optimal treatment strategy for PCNSL. High-dose methotrexate (HDMTX) is considered the cornerstone of induction therapy for PCNSL [1, 4]. Various HDMTX-based combination regimens, such as rituximab-HDMTX-cytarabine (R-MA) [5], rituximab-HDMTX-vincristine-procarbazine (R-MVP) [6], rituximab-HDMTX-temozolomide (R-MT) [7], and HDMTX-cytarabine-thiotepa-rituximab (MATRix) [5], have been extensively evaluated. Despite overall response rates (ORR) of 35–74%, their long-term control rates still fall short of expectations. Treatment-related hematologic toxicity is also a major concern, especially in the context of the COVID-19 pandemic [8]. Developing novel strategies that are more effective and less toxic is of significant clinical importance in the treatment of PCNSL.

Aberrant activation of nuclear factor kappa-B (NF-κB), an oncogenic hallmark of PCNSL, is driven by signaling pathways of B-cell receptor (BCR) and Toll-like receptor (TLR) [9]. Bruton’s tyrosine kinase (BTK) is a reasonable target for PCNSL as it links BCR and TLR to NF-κB. Ibrutinib, a first-generation BTK inhibitor (BTKi), has shown antitumor activity in patients with relapsed/refractory (R/R) PCNSL as monotherapy or combination therapy [10–12]. However, its safety profile has been compromised by off-target effects [13]. Orelabrutinib is a second-generation BTKi that offers more selective kinase inhibition and fewer off-target side effects [14]. It also has enhanced BBB permeability and bioavailability. Studies have reported that the cerebrospinal fluid (CSF) concentration of orelabrutinib was 20.10 ± 14.70 ng/mL, which is much higher than that of ibrutinib (0.77 ng/mL) [15]. Clinical data also showed that orelabrutinib could enhance the antitumor activity of rituximab [16]. Based on these findings, we propose the combination of rituximab and HDMTX with orelabrutinib (hereafter abbreviated as R-MO) as a potential regimen for patients newly diagnosed with PCNSL. The preliminary results from our phase II trial, which evaluates the safety and efficacy of the R-MO regimen, are presented here.

## Materials and methods

### Study design and participants

This is an open-label, single-arm, and multicenter investigator-initiated phase II trial. The study was registered in the Chinese Clinical Trial Registry on August 2nd, 2021 (registry number, ChiCTR2100049483) and approved by the Chinese Clinical Trial Register Ethics Committee on September 18th, 2021 (approval number, ChiECRCT20210383). All the procedures complied with the tenets of the Declaration of Helsinki, and all participants provided written informed consent.

All recruited patients met the following inclusion criteria (Supplementary Table 1): (1) newly diagnosed, histologically confirmed PCNSL (large B cell lymphoma); (2) an Eastern Cooperative Oncology Group performance status (ECOG PS) score of 0–2 (ECOG PS 3–4 permitted if due to neurological deficits); (3) age of 18 years or older; (4) life expectancy of at least 12 weeks; (5) use of effective contraception for the duration of the study and for 90 days after the last treatment if of childbearing/fathering potential; and (6) adequate bone marrow, renal, and hepatic functions. Patients with immunodeficiency were excluded.

### Treatment schedule

The trial consists of five phases: 5–15 days of initial screening, 18 weeks of induction therapy, approximately one month of high-dose chemotherapy and an autologous stem cell transplantation (ASCT) phase, depending on patient’s tolerance and preference, followed by two years of maintenance therapy, and ongoing follow-up to monitor for late recurrence.

During the induction phase, all patients received the R-MO regimen, which consisted of a rituximab dose of 375 mg/m^2^ administered intravenously on day 1 and 3.5 g/m^2^ of methotrexate intravenously on Day 2 for 3 hours. After 24 h of HDMTX infusion, leucovorin calcium salvage therapy was administered intravenously at 10–15 mg/m^2^ every six hours for a total of eight doses until the serum methotrexate concentrations dropped below 0.05 μmol/L. Urine output was closely monitored and maintained at 3000 mL/24 h, and patients are hydrated and alkalinized urine before and after HDMTX treatment. Once methotrexate levels normalize, oral orelabrutinib was given at 150 mg once daily. The induction cycle was repeated every three weeks for a maximum of 6 cycles until disease progression (PD) or intolerable toxicity.

Following induction immunochemotherapy, responders (≥partial response [PR]) continued to receive consolidation/maintenance treatment, while non-responders were offered salvage therapy. Responders who met transplant eligibility criteria—including age ≤70 years, adequate organ function (e.g., cardiac, hepatic, renal), ECOG PS ≤ 2, adequate autologous peripheral blood stem-cell collection, and absence of persistent iatrogenic side-effects—had the option to undergo high-dose chemotherapy and ASCT. Subsequently, oral orelabrutinib maintenance therapy (150 mg, oral, once daily) was initiated until disease progression, intolerable toxicity, withdrawal of consent, or two years after completion of maintenance treatment. Responders who are not eligible for transplantation or who refuse transplantation can receive orelabrutinib monotherapy maintenance therapy directly.

### Study endpoints and assessments

Pretreatment evaluation comprised of medical history, physical examination, laboratory tests, bone marrow aspiration and biopsy, positron emission tomography (PET)/computed tomography (CT) scans, cranial enhanced magnetic resonance imaging (MRI) with gadolinium enhancer, CSF cytology with flow cytometry, and ophthalmologic examination. Efficacy assessments were performed after three cycles of induction therapy, at the end of induction therapy, and every six months thereafter. Starting five years after completion of treatment, patients were scheduled for annual imaging until death or loss to follow-up. Tumor response was evaluated by gadolinium-enhanced brain MRI in accordance with the modified International PCNSL Collaborative Group Response Criteria, with categories including complete remission (CR), PR, stable disease (SD), or PD. Concurrent whole-body PET/CT or enhanced CT was conducted to exclude peripheral recurrence. If disease progression was suspected, imaging evaluations were performed earlier than the protocol-specified time points. At each visit, the following parameters were routinely collected: ECOG PS, vital signs, complete physical and neurologic examination, laboratory tests, and AEs.

The primary endpoint of the trial was the rate of progression-free survival (PFS) at one year, defined as the proportion of patients alive without evidence of disease relapse or progression one year after the initiation of therapy. Secondary efficacy outcome metrics included ORR (defined as the proportion of patients achieving CR or PR), complete remission rate (CRR, defined as the proportion of patients achieving CR), disease control rate (DCR, defined as SD or better), disease-specific survival (DSS, defined as the time from intervention to death from PCNSL), overall survival (OS, defined as the time from intervention to death, regardless of cause), and activities of daily living (ADL, assessed by the Barthel Index). Efficacy and survival analyses were conducted on the evaluable analysis set, which included all patients who received at least one dose of R-MO and had at least one efficacy assessment after the first infusion. Outcomes were also determined across multiple prespecified subgroups.

Safety was also a secondary endpoint and was assessed by evaluating the incidence of adverse events (AEs) in patients, graded according to the Common Terminology Criteria for Adverse Events (version 5.0). When reporting the overall incidence of AEs, we included each patient only once, even if they experienced the same event multiple times, and recorded the highest grade of the event to calculate its frequency. All patients who received at least one dose of therapy were considered evaluable for safety.

### Sample size calculation

Sample size calculations were conducted using PASS, version 15.0.5 (NCSS Statistical Software). Historical data indicate that HDMTX plus rituximab resulted in a 1-year PFS rate of approximately 45%, while methotrexate-cytarabine plus rituximab resulted in a 1-year PFS rate of 55% [5, 17]. Therefore, we assumed a 1-year PFS rate of 50% in the historical control group, whereas a 1-year PFS rate of ≥75% was considered to be promising in the test group. With the assumption of 10% loss to follow-up, the trial required a sample size of 37 patients to achieve 85% statistical power at a two-sided alpha level of 0.05.

### Statistical analysis

Descriptive statistics were employed to quantify medians and ranges for continuous variables and numbers and percentages for categorical variables. Response rates were summarized with point estimates and 95% exact confidence intervals (CIs) using binomial distribution. The median follow-up time was calculated using the reverse Kaplan–Meier (KM) method. Survival was estimated using the KM method. The Log-rank (Mantel–Cox) method was employed to assess the statistical significance of differences in survival curves. Sankey diagrams were constructed to illustrate the relationship between efficacy and outcomes using the R package ggalluvial. All analyses were conducted using R software (version 4.2.1). A two-sided *P* value of less than 0.05 indicated statistical significance.

## Results

### Patient recruitment

As shown in Supplementary Fig. 1, a total of 42 patients with DLBCL-subtype PCNSL were initially identified from August 2021 to July 2024. However, five patients were excluded from the study due to testicular involvement (*n* = 2) and ECOG PS of 4 (*n* = 3). As a result, the final study cohort consisted of 37 patients.

### Patient characteristics

Table 1 summarizes the baseline characteristics of the 37 patients treated with the R-MO regimen. The median age of the patients was 62 years (range, 27–78), with 51.4% being female. Of the patients, 67.6% had non-germinal center B-cell (GCB) DLBCL, while 32.4% had double-expressor lymphoma. A total of 62.2% of patients exhibited multiple foci, including two cases with leptomeningeal involvement, one case with spinal cord involvement, and one case with intraocular involvement. Additionally, 73% of patients presented with lesions involving deep structures (defined as periventricular regions, basal ganglia, corpus callosum, brainstem, or cerebellum). Elevated lactate dehydrogenase was observed in 16.2% of patients, while elevated CSF protein was observed in 35.1%. Two patients had an ECOG PS score of 3 due to neurological deficits. According to the IELSG scoring system, 73% of patients were classified as intermediate risk, while 10.8% were categorized as high risk. Using the MSKCC scoring system, 43.2% of patients were designated as intermediate risk, and 43.2% were classified as high risk. About one-third of patients underwent stereotactic or open biopsy of intracranial lesions, while the remainder underwent maximal safe resection (aimed at optimal cytoreduction while preserving neurological function).

**Table 1:** Baseline characteristics of participants.

Characteristics	All (*N* = 37)
Median age, year, median (range)	62 (27, 78)
Age, year, *n* (%)	
≤60	16 (43.2)
>60	21 (56.8)
Sex, *n* (%)	
Male	18 (48.6)
Female	19 (51.4)
Pathological types, *n* (%)	
Non-GCB DLBCL	25 (67.6)
GCB DLBCL	12 (32.4)
Double-expressor lymphomas^a^, *n* (%)	
Yes	12 (32.4)
No	25 (67.6)
Ki67, %, median (range)	80 (60, 95)
Multiple lesions, *n* (%)	
Yes	23 (62.2)
No	14 (37.8)
Deep brain lesions^b^, *n* (%)	
Yes	27 (73)
No	10 (27)
Concurrent sites, *n* (%)	
Leptomeningeal	2 (5.4)
Spinal cord	1 (2.7)
Intra-ocular	1 (2.7)
Lactate dehydrogenase, *n* (%)	
Elevated	6 (16.2)
Normal	31 (83.8)
CSF Protein, *n* (%)	
Elevated	13 (35.1)
Normal	24 (64.9)
ECOG PS, *n* (%)	
1	14 (37.8)
2	21 (56.8)
3	2 (5.4)
IELSG scoring system, *n* (%)	
Low risk	6 (16.2)
Intermediate risk	27 (73)
High risk	4 (10.8)
MSKCC scoring system, *n* (%)	
Low risk	5 (13.5)
Intermediate risk	16 (43.2)
High risk	16 (43.2)
Diagnosis modality, *n* (%)	
Stereotactic/open biopsy of intracranial lesions	14 (37.8)
Maximal safe resection of intracranial lesions	23 (62.2)

*GCB* germinal center B-cell-like, *DLBCL* diffuse large B-cell lymphoma, *CSF* cerebrospinal fluid, *ECOG PS* Eastern Cooperative Oncology Group performance status, *IELSG* International Extranodal Lymphoma Study Group, *MSKCC* Memorial Sloan-Kettering Cancer Center.

^a^Double-expressor lymphomas were defined as ≥40% MYC expression and ≥50% BCL-2 expression in tumor cells, as assessed by immunohistochemistry.

^b^Deep brain lesions were defined as those involving the periventricular zone, basal ganglia, corpus callosum, brainstem, and cerebellum.

### Efficacy

**Fig. 1:**
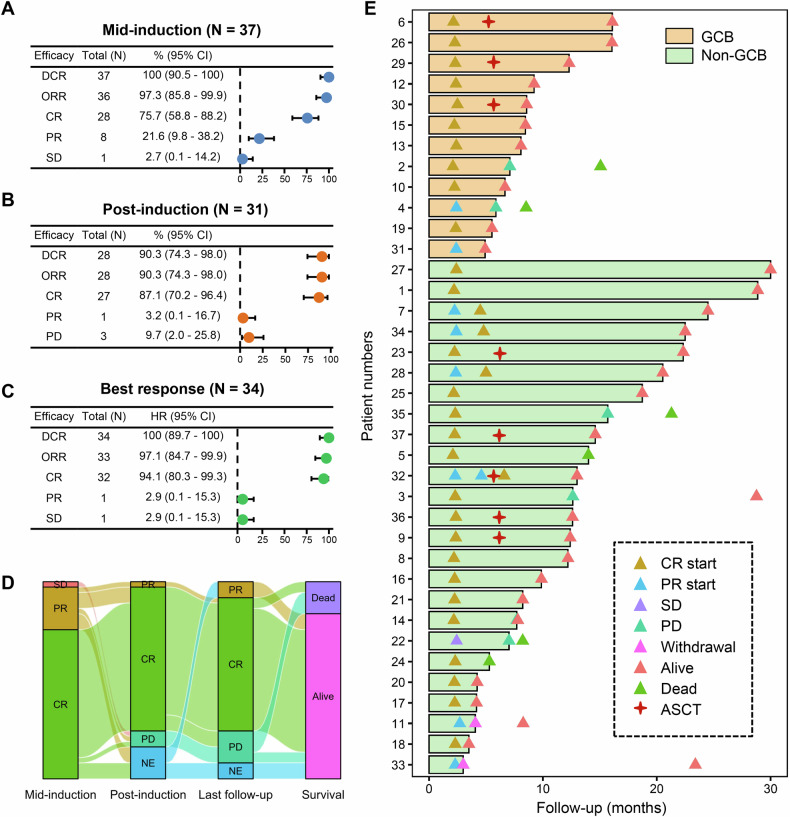
Efficacy analysis. **A** Response rates at mid-induction; **B** Response rates at the end of induction therapy; **C** The best response rates recorded throughout the trial; **D** A Sankey diagram illustrating the relationship between treatment efficacy and patient outcomes; and **E** Swim-lane plots showing the duration of treatment and corresponding responses. Note: Efficacy assessments at the end of induction therapy were inconclusive for six patients. Five of these patients had not yet finished their induction regimen, while one patient discontinued prematurely due to dissatisfaction with the PR achieved. The best response observed during the trial was ambiguous for three patients. Two patients, who had achieved a PR at the mid-induction evaluation, were still in treatment with the possibility of achieving a CR remaining uncertain. One patient withdrew from the study prematurely due to dissatisfaction with the PR, which was an unplanned event. CI confidence interval, DCR disease control rate, ORR overall response rate, CR complete remission, PR partial remission, SD stable disease, PD progressive disease, NE not evaluable, GCB germinal center B-cell-like, ASCT autologous hematopoietic stem cell transplantation.

All enrolled subjects underwent at least one efficacy assessment. Figure 1A–E depicts the treatment responses, with 36 out of 37 patients (97.3%) demonstrating a response by mid-induction. Within this cohort, 28 patients achieved CRs (75.7%) and 8 patients achieved PRs (21.6%). Upon completion of induction therapy, 31 out of 37 patients were eligible for efficacy evaluation. Of these, 28 patients successfully completed the full six cycles of planned induction therapy; however, the remaining three patients discontinued treatment before completing cycle 6 due to disease progression. The post-induction ORR was 90.3% (95% CI, 74.3–98.0), with a CRR of 87.1% (95% CI, 70.2–96.4). It is noteworthy that all progressive lesions observed during induction were localized to CNS. At the time of this report, the best observed ORR throughout the trial was 97.1% (95% CI, 84.7–99.9), and the best CRR was 94.1% (95% CI, 80.3–99.3). The forest plot (Supplementary Fig. 2) demonstrates consistently high CR rates across molecular and clinical subgroups, including cell-of-origin (GCB/non-GCB), double-expressor status, and risk stratification (IELSG/MSKCC). Notably, CR rates generally increased from mid-induction to end-of-induction, highlighting the importance of completing the full treatment cycle.

### Survival

**Fig. 2:**
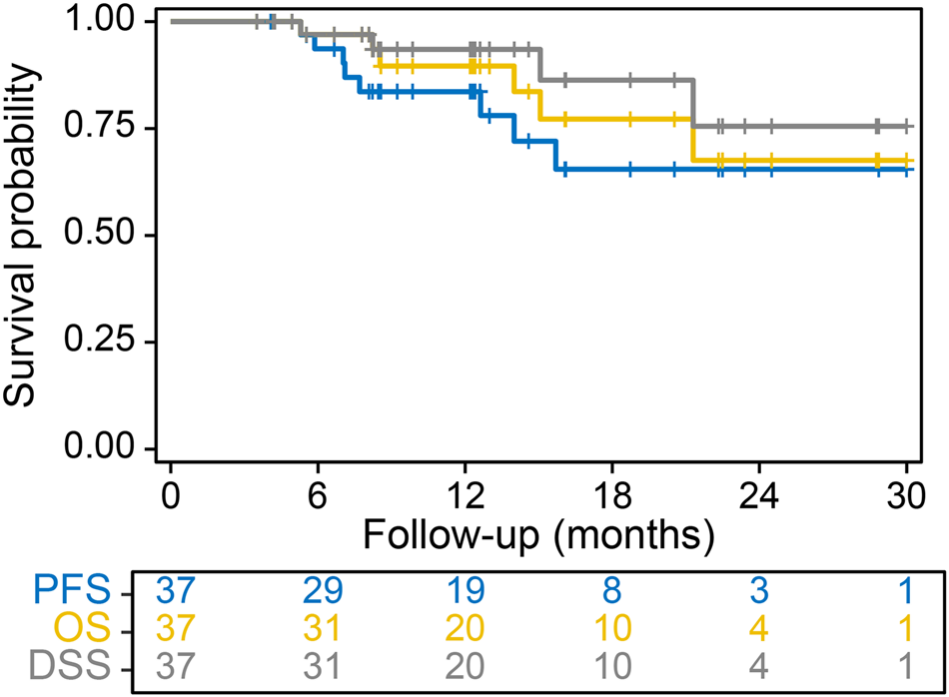
Kaplan–Meier survival curves of efficacy-evaluable population. PFS progression-free survival, OS overall survival, DSS disease-specific survival.

The median follow-up was 12.6 months (95% CI, 9.2–20.5 months). Figure 2 illustrates the KM survival curves for the efficacy-evaluable population. The median PFS was not reached, with a 6-month PFS rate of 93.6% (95% CI, 85.5–100%) and a 1-year PFS rate of 83.6% (95% CI, 71.4–97.9%), meeting the primary study endpoint. The median OS and DSS were not reached. At 6 months, the OS rate was 93.5% (95% CI, 85.2–100%) and the DSS rate was 97% (95% CI, 85.2–100%). At one year, the OS rate was 89.6% (95% CI, 69.6–100%) and the DSS rate was 93.5% (95% CI, 85.2–100%).

**Fig. 3:**
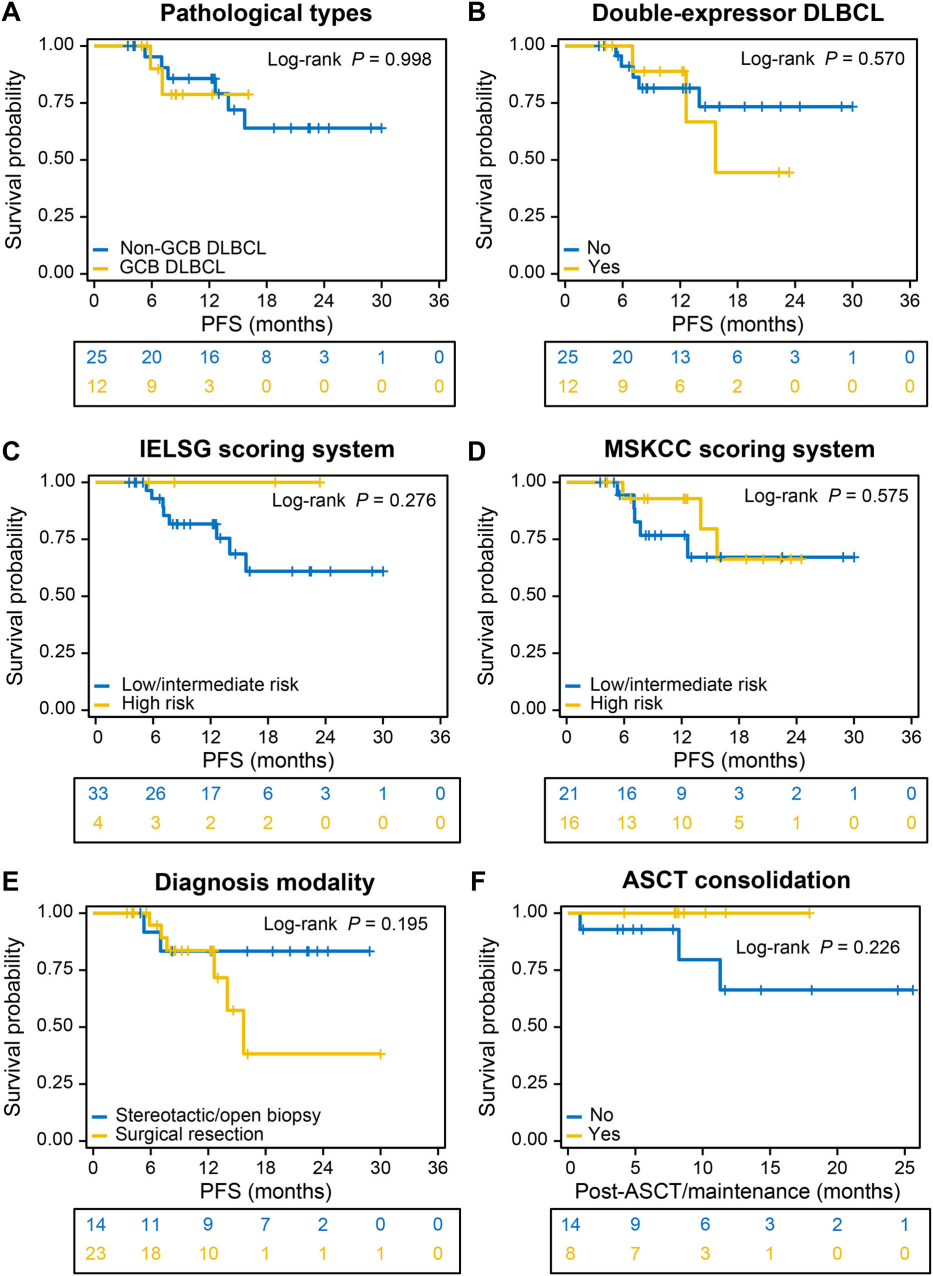
Subgroup analyses of progression-free survival in the efficacy-evaluable population. Survival curves are stratified by: **A** pathological types, **B** double-expression lymphoma status, **C** IELSG prognostic score group, **D** MSKCC prognostic score group, **E** diagnostic modality, and **F** receipt of ASCT consolidation therapy. Note: PFS in panel (**F**) is defined as the time from the initiation of either ASCT consolidation or orelabrutinib maintenance therapy to disease relapse/progression or death from any cause, whichever occurs first. GCB germinal center B-cell-like, DLBCL diffuse large B-cell lymphoma, PFS progression-free survival, IELSG International Extranodal Lymphoma Study Group, MSKCC Memorial Sloan-Kettering Cancer Center, ASCT autologous hematopoietic stem cell transplantation.

Figure 3 depicts subgroup analyses on PFS in the efficacy-evaluable population. The R-MO regimen appeared to alleviate the negative prognostic impact of the non-GCB and double-expressor subtypes (Fig. 3A, B). Patients with the non-GCB and GCB subtypes demonstrated 1-year PFS rates of 85.7% (95% CI, 72–100%) and 78.8% (95% CI, 56.4–100%), respectively, with neither median PFS being reached. The 1-year PFS rate for patients with double-expressor DLBCL was 88.9% (95% CI, 70.6–100%), which is slightly higher than that of non-double-expressor patients, at 81.5% (95% CI, 66.6–99.7%). The median PFS for patients with double-expressor DLBCL was 15.7 months. Patients categorized as low/intermediate risk according to the IELSG and MSKCC systems exhibited 1-year PFS rates as high as 81.7% (95% CI, 68.4–97.6%) and 76.7% (59.1–99.6%), respectively (Fig. 3C, D). For high-risk patients classified in the same manner, 1-year PFS rates were 100% for the IELSG system and 92.9% (95% CI, 80.3–100%) for the MSKCC system. Compared with biopsy, maximal safe resection of the lesion did not appear to improve patient outcomes (Fig. 3E, *P* = 0.195).

A total of 22 patients met the criteria for ASCT after induction. Among them, 8 patients chose the consolidation regimen of ASCT followed by maintenance with Orelabrutinib, and 8 patients refused ASCT and directly entered the maintenance phase. One patient was in PR before transplant, but achieved a CR subsequent to transplant. After a median follow-up of 8.6 months (range, 7.8–17.9 months), all patients who underwent transplanted maintained CR status, resulting in a 1-year PFS rate of 100%. In the untransplanted group (*n* = 14), the 1-year PFS rate was 66.3% (95% CI, 40.6–100%). There was no significant difference in post-ASCT/maintenance remission duration between the two groups (Fig. 3F, *P* = 0.226).

### Quality-of-life

Following just one treatment cycle, there was a marked improvement in the quality of life for the responders, as evidenced by a significant increase in the median Barthel Index from 70 (range 0–95) to 90 (range 55–100), with a *P*-value of 0.024 (Supplementary Fig. 3). This enhancement was maintained, with the median Barthel Index remaining at 95 both at the mid-point and the end of the induction phase.

### Safety

All patients were assessable for toxicity. Table 2 summarizes the incidence of all-cause AEs. Neutropenia, lymphocytopenia, and infections were the most prevalent AEs, affecting 45.9% of patients each. Frequent grade 3–4 AEs included neutropenia (43.2%), infections (29.7%), and febrile neutropenia (29.7%). Two patients (5.4%) developed grade 5 pulmonary infections. Despite occurring during the remission phase, due to the immunosuppressive effects of the BTKi and rituximab, we classify these two cases of pulmonary infections as potentially related. Treatment delays occurred in four patients due to AEs including febrile neutropenia, acute kidney injury, infusion reactions, and liver function impairment. In four patients (10.8%), the methotrexate dose was reduced to 3 g/m^2^ because of kidney function impairment. No patients discontinued treatment permanently due to AEs.

**Table 2:** Treatment-emergent adverse events (*N* = 37).

CTCAE term	All	G1-2	G3-4	G5
	No. (%)	No. (%)	No. (%)	No. (%)
Neutrophil count decreased	17 (45.9)	1 (2.7)	16 (43.2)	–
Lymphocyte count decreased	17 (45.9)	8 (21.6)	9 (24.3)	–
Infection event	17 (45.9)	4 (10.8)	11 (29.7)	2 (5.4)^a^
Anemia	16 (43.2)	13 (35.1)	3 (8.1)	0
Fever	16 (43.2)	13 (35.1)	3 (8.1)	0
White blood cell decreased	15 (40.5)	5 (13.5)	10 (27)	–
Platelet count decreased	12 (32.4)	4 (10.8)	8 (21.6)	–
Lung infection	12 (32.4)	3 (8.1)	9 (24.3)	0
Febrile neutropenia	11 (29.7)	–	11 (29.7)	0
Alanine aminotransferase increased	9 (24.3)	8 (21.6)	1 (2.7)	–
Alkaline phosphatase increased	9 (24.3)	8 (21.6)	1 (2.7)	–
Cough	9 (24.3)	2 (5.4)	7 (18.9)	–
Aspartate aminotransferase increased	8 (21.6)	5 (13.5)	3 (8.1)	–
Gamma-glutamyl transpeptidase increased	8 (21.6)	8 (21.6)	0	–
Hypokalemia	8 (21.6)	5 (13.5)	3 (8.1)	0
Fatigue	8 (21.6)	8 (21.6)	0	–
Dizziness	8 (21.6)	4 (10.8)	4 (10.8)	–
Hypocalcemia	7 (18.9)	7 (18.9)	0	0
Bleeding event	7 (18.9)	5 (13.5)	2 (5.4)	0
Creatinine increased	5 (13.5)	2 (5.4)	3 (8.1)	–
Diarrhea	5 (13.5)	5 (13.5)	0	0
Blood bilirubin increased	4 (10.8)	4 (10.8)	0	–
Acute kidney injury	4 (10.8)	–	4 (10.8)	0
Hyponatremia	4 (10.8)	1 (2.7)	3 (8.1)	0
Headache	4 (10.8)	1 (2.7)	3 (8.1)	–
Nausea	4 (10.8)	4 (10.8)	0	–
Infusion reactions to rituximab	4 (10.8)	2 (5.4)	2 (5.4)	0
Hyperglycemia	3 (8.1)	1 (2.7)	2 (5.4)	0
Vomiting	3 (8.1)	3 (8.1)	0	0
Atrial fibrillation	1 (2.7)	0	1 (2.7)	0

Note: “0” indicates no events within CTCAE v5.0 categories; “–” denotes unavailable grading.

^a^Both grade 5 events were lung infections, with one developing secondary sepsis.

### Cause of death

By November 2024, six patients had died. Four fatalities were due to disease progression, with a median OS of 11.3 months (range: 5.3–21.3 months). The other two deaths, attributed to pulmonary infections during CR, had median OS of 8.53 and 14 months, respectively.

## Discussion

Our study highlights the novel R-MO regimen’s efficacy in newly diagnosed PCNSL, achieving a 1-year PFS rate of 83.6% and an ORR of 97.1% at a median follow-up of 12.6 months. Previous studies report 1-year PFS rates of 57–79% for HDMTX-rituximab regimens (e.g., MATRix) and <60% for HDMTX-based multi-drug chemotherapy (e.g., MVP) [18]. Our data surpasses historical benchmarks, primarily attributed to the integration of BTKis during the induction phase. This approach overcomes the limitations of traditional chemotherapy, highlighting the pivotal role of BTKis in optimizing induction therapy for improved clinical outcomes. Besides, historical regimens (e.g., MATRix) were burdened by toxicities like severe infections (11–28% per cycle, with 6% requiring intensive care unit admission) [19]. In contrast, our protocol maintained efficacy while reducing toxicity, underscoring its clinical viability.

Current evidence supports 3.5 g/m² as an effective dose for PCNSL. Wang et al. [20] demonstrated comparable efficacy with reduced toxicity at 3–5 g/m² compared to higher doses (>5 g/m²), a finding corroborated by Dalia et al. [21] showing equivalent PFS/OS between 3.5 g/m² and 8 g/m². These findings suggest that dose reduction strategies may improve treatment tolerance and induction completion rates, particularly in elderly patients or those with comorbidities, without compromising therapeutic efficacy. While CSF penetration of rituximab remains controversial, clinical data consistently supports its therapeutic value [22–25]. Single-agent rituximab achieved 42% ORR in relapsed/refractory PCNSL [25]. The IELSG32 trial revealed superior ORR (74% vs 53%) and PFS with R-MA versus MA alone [5], supported by Schmitt et al’ meta-analysis demonstrating PFS improvement despite OS neutrality [26]. Considering the synergy between standard methotrexate intervals (10 days to 3 weeks) and rituximab’s pharmacokinetics (median terminal half-life: 22 days), we adopted a triweekly regimen to optimize efficacy-toxicity balance. Our cumulative dose (21 g/m²) aligns with established R-MIV (21 g/m²) and R-MPV (17.5–24.4 g/m²) protocols [5, 6]. The observed bone marrow toxicity profile (30% incidence of febrile neutropenia) likely results from synergistic myelosuppression induced by triple-agent interactions and potential subclinical COVID-19 viral insults during the pandemic period. Based on this, we recommend against shortening the treatment cycle to 14 days due to potential exacerbation of myelosuppressive toxicity.

Targeting the BCR/NF-κB pathway with BTKis has led to a breakthrough in the treatment of PCNSL. While the first-generation BTKi ibrutinib is hindered by high side effects, the second-generation tirabrutinib is approved for R/R PCNSL in Japan [13, 27, 28]. Our country’s novel BTKi, orelabrutinib, boasts high BBB permeability, excellent bioavailability, and selective kinase targeting [14]. In a retrospective study by Yang et al. [29], chemotherapy involving orelabrutinib in 15 R/R PCNSL patients showed an 86.7% response rate, with a 73.3% CRR and favorable safety. A phase II trial combining orelabrutinib and programmed death protein 1 inhibitors sans chemotherapy in R/R PCNSL patients achieved a 61.5% ORR and an estimated 66.7% 1-year PFS rate [30]. Various ongoing clinical trials, such as NCT05021770, NCT05390749, and NCT05036577, are exploring orelabrutinib-based regimens for PCNSL. This pioneering study marks the first report on orelabrutinib-based regimen treatment for newly diagnosed PCNSL patients, with long-term follow-up results eagerly awaited. To further validate BTKi’s role, we recommend a phase III randomised controlled trial comparing BTKi-containing induction/maintenance versus standard HDMTX-based regimens, with PFS as primary endpoint and comprehensive safety monitoring.

Upon completion of methotrexate-based treatment, consolidation strategies are often employed to prolong the response duration. However, despite these efforts, PCNSL has a high rate of recurrence, particularly in the first two years after treatment [3]. Based on this recurrence window, we structured the maintenance protocol to administer orelabrutinib for 24 months to all responding patients, regardless of whether they had undergone ASCT. It is worth noting that assessing the clinical value of orelabrutinib maintenance therapy is premature, as the majority of patients have not completed two years of maintenance therapy. Additionally, another randomized phase III trial (NCT05334238) is currently investigating the efficacy and safety of orelabrutinib maintenance post-ASCT in PCNSL. The necessity of ASCT consolidation remains uncertain; a randomized trial comparing ASCT consolidation (without maintenance) versus BTKi maintenance alone would definitively address its clinical value.

Our phase II results are promising, yet the limited number of enrolled patients leaves room for interpretation of the results. Our analyses included only Chinese patients, underscoring the need for further validation in larger, more diverse, and multiethnic populations. Additionally, extended follow-up is essential to assess long-term outcomes and effects of the R-MO regimen on PCNSL patients.

## Conclusions

With a 1-year PFS rate of 83.6%, the study met its primary end point. The incorporation of orelabrutinib to HDMTX and rituximab in newly diagnosed PCNSL patients has shown promising outcomes and a favorable safety profile. Further follow-up is required to fully evaluate the maintenance value of orelabrutinib. Future prospective phase III multicenter trials will be essential to validate the findings of this study.

## Supplementary information

Supplementary Materials

## Data Availability

The datasets analyzed during the current study are available from the corresponding authors on reasonable request.
