# Supplementary material for: Prospective phase II trial of first-line rituximab, methotrexate, and orelabrutinib (R-MO) in primary central nervous system lymphoma
Source: Blood Cancer J. 2025 Apr 29;15(1):81. doi: 10.1038/s41408-025-01278-w (PMC12041229; doi:10.1038/s41408-025-01278-w)
Supplement: Supplementary file 1 — Supplementary Materials [file 41408_2025_1278_MOESM1_ESM.docx]

**Supplementary Materials**

**Title:** Prospective phase II trial of first-line methotrexate, rituximab, and orelabrutinib (R-MO) in primary central nervous system lymphoma

**Authors:** Lixia Sheng^1,*^, Hailing Liu^2,3,*^, Xiaohui Zhang^4,*^, Kaiyang Ding^5^, Jie Ma^6^, Hongling Peng^7^, Xia Zhao^8^, Mei Sun^9^, Wei Shi^10^, Feiyan Zhang^2,3^, Jianyong Li^2,3^, Lei Cao^2,3,†^, and Lei Fan^2,3,†^

^*^ These authors contributed equally to this work

^†^ **Corresponding authors:** Lei Fan, M.D., Ph.D. (first corresponding author), Lei Cao, M.D. (co-corresponding author)

E-mail: fanlei@jsph.org.cn (Lei Fan); [woshicaolei.2008@163.com](mailto:woshicaolei.2008@163.com) (Lei Cao)

**This file includes:**

**Supplementary Table 1.** Inclusion and exclusion criteria

**Supplementary Figure 1.** Flow chart of study design

**Supplementary Figure 2.** Response rates by subgroups during induction therapy

**Supplementary Figure 3.** Quality-of-life score analyses of responders

**Supplementary Table 1.** Inclusion and exclusion criteria

| **Inclusion Criteria** | 1. Newly diagnosed, histologically confirmed PCNSL (large B cell lymphoma); 2. An ECOG PS score of 0-2 (ECOG PS 3-4 permitted if due to neurological deficits); 3. Age of 18 years or older; 4. Life expectancy of at least 12 weeks; 5. Use of effective contraception for the duration of the study and for 90 days after the last treatment if of childbearing/fathering potential; 6. Adequate bone marrow, renal, and hepatic functions: 7. Complete blood count: absolute neutrophil count ≥ 1.0×10^9^/L, platelets ≥50×10^9^/L, hemoglobin ≥7.0g/dL; 8. Hepatic function: total bilirubin ≤2×ULN, alanine aminotransferase ≤3×ULN, aspartate aminotransferase ≤3×ULN; 9. Renal function: serum creatinine ≤2×ULN; 10. Signed the informed consent form and able to comply with the scheduled follow-up visits and related procedures required in the protocol. |
| --- | --- |
| **Exclusion Criteria** | 1. Pregnant or lactating patients; 2. Patients who have contraindications for any drug involved in the program; 3. Patients enrolled in another interventional clinical study; 4. Received major surgery within two weeks before the first dose of study medication, or have unhealed wounds, ulcers, or fractures; 5. Received any live attenuated vaccine within four weeks before the first dose of study medication, or is scheduled to receive the live attenuated vaccine during the study period; 6. Uncontrolled concurrent diseases, including but not limited to: 7. HIV-infected patients; 8. Active pulmonary tuberculosis; 9. Acute and chronic active hepatitis; 10. Symptomatic congestive heart failure (NYHA Class III-IV) or symptomatic or poorly controlled arrhythmia; 11. Any thromboembolic events that occurred within three months before enrollment; 12. Any life-threatening bleeding events that occurred within three months before enrollment; 13. Acute or chronic diseases, psychiatric disorders, or laboratory abnormalities that may lead to increased investigational drug-related risks, or interference with interpreting trial results, and are considered by the investigators to make the patient ineligible for participating in the trial; 14. Inability to understand or follow research protocols. |

Abbreviations: PCNSL, primary central nervous system lymphoma; ECOG PS, Eastern Cooperative Oncology Group performance status; ULN, upper limit of normal; NYHA, New York Heart Association


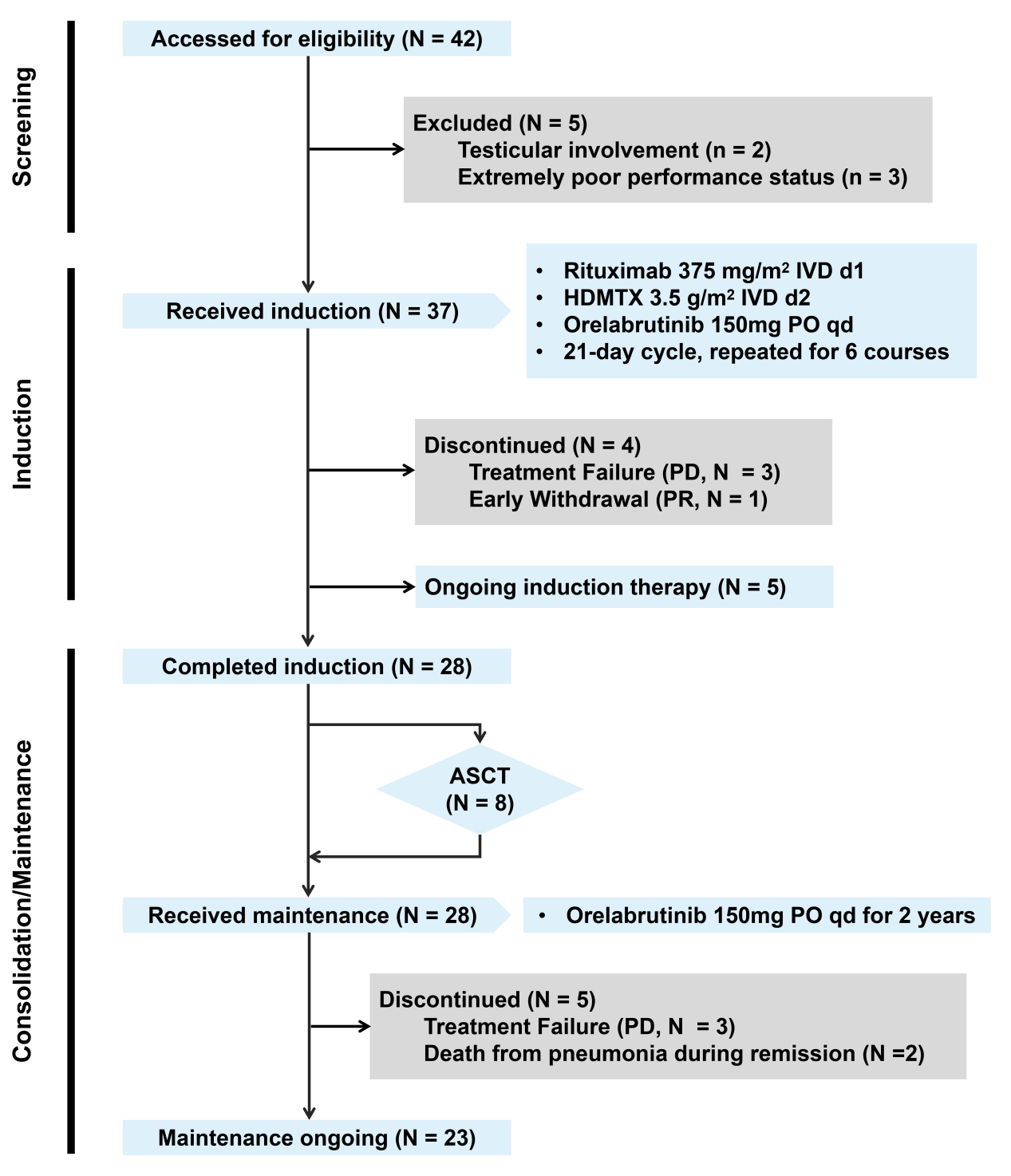


**Supplementary Figure 1.** Flow chart of study design

Abbreviations: HDMTX, high-dose methotrexate; PD, disease progression; PR, partial remission; ASCT, autologous stem cell transplantation.


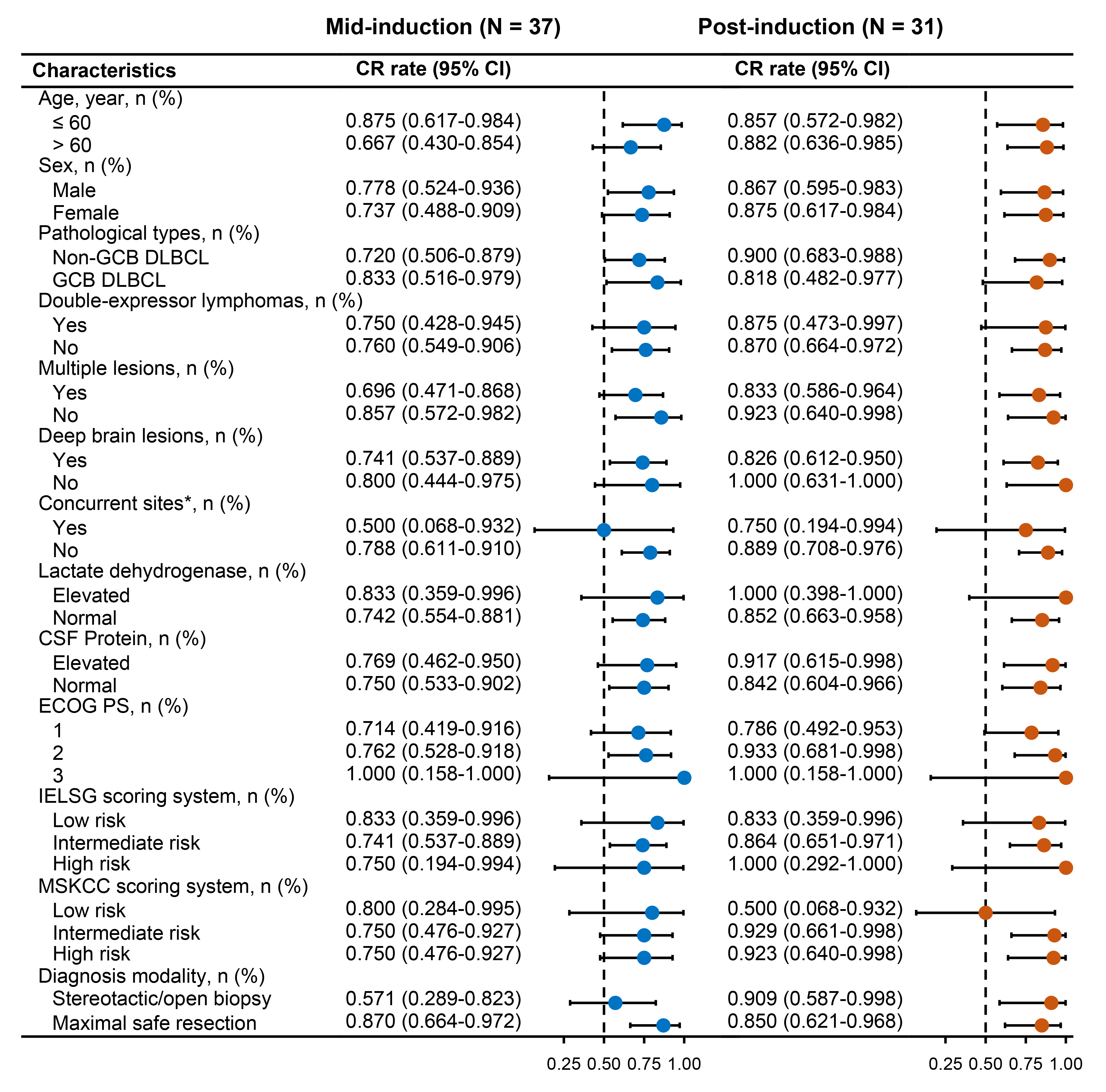


**Supplementary Figure 2.** Response rates by subgroups during induction therapy

"*" refers to leptomeningeal, spinal cord, or intraocular involvement. p-values in italics and bold display statistically significant results.

Abbreviations: CR, complete response; CI, confidence interval; GCB, germinal center B-cell-like; DLBCL, diffuse large B-cell lymphoma; CSF, cerebrospinal fluid; ECOG PS, Eastern Cooperative Oncology Group performance status; IELSG, International Extranodal Lymphoma Study Group; MSKCC, Memorial Sloan-Kettering Cancer Center.


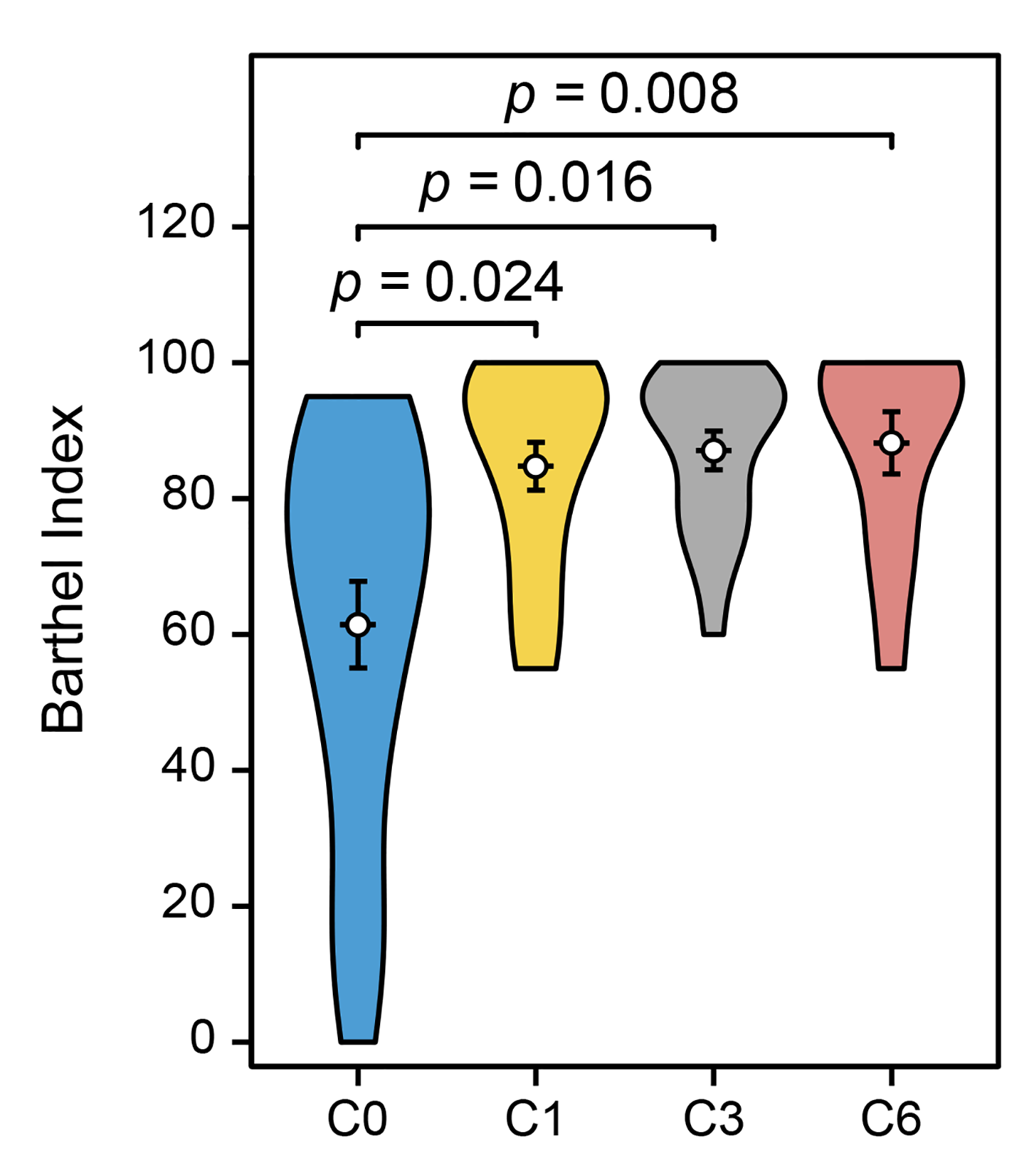


**Supplementary Figure 3.** Quality-of-life score analyses of responders
